# Revisiting the Impact of Environmental Regulation on Green Total Factor Productivity in China: Based on a Comprehensive Index of Environmental Regulation from a Spatiotemporal Heterogeneity Perspective

**DOI:** 10.3390/ijerph20021499

**Published:** 2023-01-13

**Authors:** Lei Jiang, Yuan Chen, Bo Zhang

**Affiliations:** 1 School of Geography and Remote Sensing, Guangzhou University, Guangzhou 510006, China; 2 Guangdong Provincial Center for Urban and Migration Studies, Guangzhou 510006, China; 3 School of Economics, Jinan University, Guangzhou 510610, China; 4 Southern Marine Science and Engineering Guangdong Laboratory (Zhuhai), Zhuhai 519000, China

**Keywords:** environmental regulation, comprehensive evaluation method, GTFP, Global Malmquist Luenberger index, GTWR, spatiotemporal heterogeneity, Porter hypothesis

## Abstract

Promoting greener and sustainable development is one of the main goals of the most recent 14th Five-Year Plan (i.e., 2021–2025). Environmental regulation is seen as fundamental to green transformation and an important way for all of China to reach a high-quality and sustainable development mode. However, large spatial disparities exist across the different regions in China, so formulating region-oriented environmental regulatory policies to achieve regional high-quality and sustainable development is now a matter of great practical significance. In the present paper, we analyze this problem and begin by calculating the high development level measured through the Green Total Factor Productivity (GTFP) of 259 Chinese cities. Thereafter we construct a comprehensive index of environmental regulation through the linear weighted-sum method. Lastly, we investigate the spatiotemporal heterogeneity of the impact of environmental regulation on GTFP using a Geographically and Temporally Weighted Regression (GTWR) model. We find that: (1) From the spatial dimension perspective, the impact of environmental regulation of Chinese cities on GTFP is either linear (monotonically increasing or decreasing), non-linear (U-shaped or inverted U-shaped), or nonsignificant. Most cities have a U-shaped relationship, indicating that environmental regulation first inhibits GTFP at the early stage, but then promotes it. There are also significant differences among cities in the turning points of environmental regulation; (2) From the time dimension perspective, the number of cities is on the rise having monotonically decreasing impacts of environmental regulation on GTFP. Furthermore, even for the same city, the relationship between the two variables shows different characteristics in different years; (3) The impact of five control variables on GTFP may also vary from one city to another over the sample period, also presenting spatiotemporal heterogeneity effects. Consequently, the formulation and implementation of environmental regulatory policies should not only adapt to local conditions but also choose reasonable and effective measures to achieve high-quality development targets.

## 1. Introduction

Recently, various environmental pollution problems in China, such as haze [1] (PM_2.5_ [2], CO_2_ emissions [3], water quality deterioration [4], and heavy metal contamination in soils [5], have aroused deep concern from the Chinese government and the public. For many years China has relied on a crude economic growth model that draws heavily on the use of natural resources. The problem worsened and, as a result, many of China’s environmental pollution indicators rank first in the world. The increasingly serious environmental pollution has damaged people’s health and even posed a threat to the country’s sustainable development [6]. Hence, the Chinese government has emphatically begun to promote eco-friendly economic and social development in all respects. Indeed, an effective green transition is one of the main goals of the 14th Five-Year Plan. However, environmental pollution is still one of the critical bottlenecks hindering the green transition and high-quality development in China. Zou et al. [7] assert that only by coordinating the balance of decision-making between rapid economic growth and the ecological environment, can China achieve its green and high-quality development targets.

At present, China’s environmental policies cannot completely solve the problems of environmental pollution while at the same time realizing its high-quality development goals. How to improve China’s environmental governance system and curb the increasingly serious environmental pollution is a key problem that China urgently needs to solve. To begin to address environmental degradation, the Chinese government set out pollutant emission reduction targets as early as 1996–2000 during the 9th Five-Year Plan [8].

However, as more stringent environmental regulation in China was aimed to curb environmental pollution, it became clear that maintaining rapid economic growth while simultaneously strengthening environmental protections would be a difficult task. At present in China, various cities across the country display remarkably uneven development levels; there are differences in resource endowment, degree of economic development, industrial structure, and environmental pollution status. In other words, a one-size-fits-all environmental regulation is not feasible; effective, city-oriented policies and measures for Chinese cities are therefore urgently needed. It is necessary to implement well-crafted and well-enforced environmental regulation tools that correspond to the local conditions of Chinese cities to achieve a “win-win” goal of the trade-off between environmental quality and high-quality development.

The main aim of this research was to quantify the spatiotemporal heterogeneity of the impact of environmental regulation on GTFP. To this end, a comprehensive index of environmental regulation including pollutant emissions and removal rates was built using the linear weighted sum method. Moreover, the GML index was applied to assess the GTFP scores of 259 Chinese cities from 2005 to 2016. Lastly, from the perspective of spatiotemporal heterogeneity, the GTWR model was adopted to quantify the impact of environmental regulation on GTFP.

## 2. Literature Review

The topic of an environmental regulation-productivity nexus has been discussed extensively in the literature, but the conclusions of these studies are controversial. Environmental regulation has been found to exert a negative or positive impact on productivity. On the one hand, environmental regulation may have a positive impact on productivity. Specifically, to improve environmental quality, governments usually tend to implement a broad range of policy tools, including environmental regulation, which inevitably impacts the production decisions of enterprises, e.g., innovation incentives. In other words, environmental regulation imposes a compliance cost on regulated enterprises. To cope with the consequences, they must stimulate innovation in a bid to improve product quality and lower costs, which in turn increases productivity [9]. As a result, well-crafted and well-enforced environmental regulation benefits both the environment and enterprises [10] because triggered innovation has been shown to offset the compliance costs caused by environmental regulation. This procedure is also known as the Porter hypothesis [11,12]. Too stringent environmental regulation may exhibit a negative impact on productivity because it poses an additional burden for regulated enterprises. Specifically, regulation compels enterprises to increase pollution abatement costs that could have been used for production, thus hindering productivity [13]. In other words, the considerably high compliance costs induced by environmental regulation are likely to weaken innovation incentives.

Numerous researchers worldwide have conducted in-depth studies using the Porter hypothesis and employing different data sets. For example, Hamamoto [14] focused on five Japanese polluting industries and examined the relationship between environmental regulation and Total Factor Productivity (TFP); conclusions showed that an increase in R&D investment stimulated by stricter environmental regulation had a positive impact on TFP. Lanoie et al. [15] studied the relationship between environmental regulation and TFP growth in the Quebec manufacturing sector; they found that the impact was negative. Becker [16] examined the impact of environmental regulation on the productivity of manufacturing plants in the US and found no significant effect on productivity with higher environmental compliance costs. Hancevic [17] evaluated the impact of the Title IV of the 1990 Clean Air Amendments on the productivity of coal-fired electricity generating units in the US and found that productivity declined between 1% and 2.5%. Wang et al. [9] employed OECD industrial sectors and examined the impact of environmental regulation on GTFP; their results revealed that the Porter hypothesis was validated in that environmental regulation had a positive impact. Santis et al. [18] investigated the environmental regulation-productivity nexus for 18 OECD countries; their findings also supported the hypothesis. Lena et al. [19] employed panel data on 13 Italian manufacturing industries and found that environmental regulation had no negative effect in most of the sample industries. From the abovementioned studies, we can conclude that the empirical findings have not yet reached a consensus.

China, the largest developing country in the world, has achieved an economic miracle. However, it has been facing a trade-off between rapid economic growth and serious environmental pollution problems. In recent years, China has paid close attention to how high-quality development comes with negative environmental consequences. GTFP, a recently developed index based on traditional TFP, is seen as an effective indicator for measuring a region’s high-quality development performance and considering negative environmental consequences. Researchers using the Porter hypothesis, including [20,21] among others, have addressed the challenge of evaluating economic performance in the face of dangerous pollution levels. The findings of the existing studies can be divided into three strands.

The first strand addresses environmental regulation as beneficial to GTFP improvements, thus supporting the Porter hypothesis. For example, Wang et al. [22] concluded that environmental regulation promoted China’s industrial GTFP growth, implying that the Porter hypothesis was confirmed. Zhou and Tang [23] took the “Action Plan Air Pollution Prevention and Control” as environmental regulation and examined its impact on the GTFP of China’s industries; they found that regulation promoted the GTFP in the air-intensive industries. Zhang [24] used the green credit regulation policy as environmental regulation and revisited the Porter hypothesis, where the findings showed that the policy significantly improved GTFP. Peng et al. [25] considered the SO_2_ Emissions Trading Pilot as a market-based environmental regulation and tested for the Porter hypothesis; results indicated that strict but flexible environmental regulation was more likely to enhance productivity. Using a data set of 59 prefecture-level cities in the Yellow River Basin in China, Mao et al. [26] concluded that environmental regulation was intensified but promoted GTFP in the Basin region. Lee et al. [27] investigated the impact mechanisms of environmental regulation on GTFP based on panel data from 30 Chinese provinces; they found that environmental regulation promoted GTFP.

The second research strand claims that environmental regulation inhibits GTFP growth. Cai and Ye [28] treated China’s new environmental regulation law as a quasi-natural experiment to examine the Porter hypothesis using a firm-level data set in China. Their results revealed that the law inhibited the TFP of enterprises. Peng [29] adopted a spatial econometric model to examine the direct and indirect impacts of environmental regulation on GTFP. The results showed that it promoted the GTFP in local regions; moreover, neighboring environmental regulation had negative spatial spillover effects on GTFP. Tang et al. [30] paid attention to a traditional environmental policy, namely, command-and-control environmental regulation, and re-examined the Porter hypothesis based on a Chinese industrial enterprise panel dataset. They concluded that it hindered the TFP of enterprises and highlighted the difficulty in achieving a win-win scenario between sustainable development and TFP growth. Li et al. [31] found that strict environmental regulation would inhibit GTFP in the Yangtze River Delta region in China. The above-mentioned studies remind us that the empirical evidence on this topic remains controversial.

The third literature strand representing the findings for the impact of environmental regulation on GTFP provides uncertain or nonlinear results. For example, Wang and Shen [32] showed that the relationship between environmental regulation and GTFP was an inverted U-shaped curve. Zhao et al. [33] found an inverted U-shaped curve between environmental regulation and the TFP of China’s carbon-intensive industries, indicating that the impact of environmental regulation changed from innovation offsets to compliance costs. Using panel data from industrial sectors of 30 provinces in China, Qiu et al. [34] concluded that the relationship between environmental regulation and GTFP was U-shaped. Hu and Wang [35] found a threshold for the impact of environmental regulation on carbon productivity based on panel data from 30 Chinese provinces.

Environmental regulation in different industries or cities may indeed exhibit different impacts on GTFP. For example, Li and Wu [36] employed panel data from 273 Chinese cities and applied a spatial Durbin model to verify the Porter hypothesis; their results revealed that environmental regulation had a positive impact on GTFP in high political attribute cities but a negative impact in lower political attribute cities. Feng et al. [37] re-examined the Porter hypothesis by applying a propensity score matching the difference-in-differences approach; they found that the impact of environmental regulation varied from the productivity level of enterprises. We still notice that the impact of environmental regulation may differ because it is heavily dependent on the type of environmental regulation. For example, Tian and Feng [38] introduced three types of environmental regulation in China. Their findings showed that command-and-control regulation could improve GTFP, market-based regulation damaged GTFP, and voluntary regulation exhibited a negative impact on GTFP.

The abovementioned studies have explored this topic widely and their findings not only help us understand how environmental regulation affects GTFP but also enrich the existing literature. The empirical findings are controversial; however, we observe that researchers have gradually begun to pay attention to the nonlinear impact and spatial effects of environmental regulation on GTFP. Nevertheless, most of the studies suffer from two shortcomings. They have used either province-level or industry-level data by applying global regression models. In other words, the impact of environmental regulation on GTFP is fixed with no spatial heterogeneity across all regions or industries. We need to emphasize that environmental regulation varies from one city, or one industry, to another due to huge differences in economic level, technological level, and industrial structure. In addition, environmental regulation also changes over time and may exhibit a time-varying impact on GTFP. In this sense, it can safely be assumed that the impact of environmental regulation on GTFP varies across regions with time. Importantly, the spatiotemporal heterogeneity should not be ignored when investigating the impact of environmental regulation on GTFP. Hence, in this study, we attend to the “place-time” aspect by employing a panel of 255 prefecture-level cities from 2005 to 2016 and applying the GTWR model to explore the spatiotemporal impacts of environmental regulation on the GTFP.

The research approach in the present paper has several advantages. As mentioned earlier, the linear model may not be able to fully explain the impacts of environmental regulation on the GTFP due to complex socioeconomic activity; instead, it may lead to biased conclusions. Our first advantage is our adoption of a nonlinear model specification. This study also incorporates the spatial heterogeneity effect and applies the GTWR model to investigate the nonlinear impacts of environmental regulation on GTFP over time. Furthermore, the estimated coefficients are geo-visualized in maps; these not only help us to identify regional differences in environmental regulation, but also at a later date to better design and implement city-oriented policies and measures for controlling environmental pollution and effectively improving GTFP in China.

## 3. Models and Variables

### 3.1. GTWR Model

As a benchmark, an ordinary least squares (OLS) model is provided first. However, the OLS model assumes no spatial differences in relationships between dependent and independent variables. In other words, the estimates of the explanatory variables are average and global [39]. Given that cities across China display wide differences in economic level, industrial structure, environmental condition and trajectory, and stringency of regulation, the impacts of environmental regulation on GTFP are likely to vary from city to city; that is to say, we are likely to observe spatial heterogeneity. Therefore, this paper has applied the GTWR model developed by Huang et al. [40]; their model guided us in the identification of the spatiotemporal heterogeneity of the impacts of environmental regulation on GTFP.

The GTWR model is presented as:

(1) $$Y_{t}=\beta_{0}\left(\mu_{i},\;\nu_{i},\;t_{i}\right)+\Sigma_{k}\beta_{k}\left(\mu_{i},\;\nu_{i},\;t_{i}\right)X_{ik}+\varepsilon_{i}$$

where *Y* and *X* represent the dependent variable and a set of the independent variables, respectively. $\left(\mu_{i},\;\nu_{i},\;t_{i}\right)$ denotes the space-time coordinates of city *i* in year *t;* specifically, the subscripts *u* and *v* are the coordinates of the city; *t* is the year. $\beta_{0}\left(\mu_{i},\;\nu_{i},\;t_{i}\right)$ is an intercept term and $\beta_{k}\left(\mu_{i},\;\nu_{i},\;t_{i}\right)$ represents the unknown coefficients of the *k_th_* explanatory variable to be estimated. Lastly, $\varepsilon_{i}$ is a stochastic disturbance term.

The estimated coefficients $\beta \left(\mu_{i},\;\nu_{i},\;t_{i}\right)$ can be obtained through a local linear estimation method expressed as:

(2) $$\hat{\beta }\left(\mu_{i},\;\nu_{i},\;t_{i}\right)={\left(X^{T}W\left(\mu_{i},\;\nu_{i},\;t_{i}\right)X\right)}^{-1}X^{T}W\left(\mu_{i},\;\nu_{i},\;t_{i}\right)Y$$

One can see from Equation (2) that the essential problem of the GTWR model is the spatial weighting function $W\left(\mu_{i},\;\nu_{i},\;t_{i}\right)$. In this study, we followed Huang et al. [40] by adopting the widely-used Gaussian function in empirical studies.

To quantify the spatial-temporal heterogeneity of the impact of environmental regulation on GTFP of each city, the GWTR model is thus built as follows:

(3) $$GTFP_{i}=\beta_{0}\left(\mu_{i},\;\nu_{i},\;t_{i}\right)+\beta_{1}\left(\mu_{i},\;\nu_{i},\;t_{i}\right)ER_{i}+\beta_{2}\left(\mu_{i},\;\nu_{i},\;t_{i}\right)ER^{2}+X_{i}\theta \left(\mu_{i},\;\nu_{i},\;t_{i}\right)+\varepsilon_{i}\left(\mu_{i},\;\nu_{i},\;t_{i}\right),$$

where GTFP is the dependent variable; *ER* is the core explanatory variable, namely, environmental regulation; *ER*^2^ is the squared term of *ER*. In this study, we examined the possibility of a nonlinear relationship between environmental regulation and GTFP by incorporating the *ER* and *ER2* variables. To avoid omitted-variables bias, we also considered a set of explanatory variables, including innovation level (*Innov*); industrial structure (*Second*); urbanization level (*Urban*); economic level (*Econ*); and educational level (*Edu*), which will be introduced in detail later.

$\beta_{1}$ and $\beta_{2}$ are two unknown parameters of the *ER* and *ER*^2^ variables to be estimated, respectively. When $\beta_{1}>0$ (or $\beta_{1}<0$) and $\beta_{2}$ is highly nonsignificant, it indicates that the relationship between *ER* and *GTFP* is monotonically increasing (or decreasing); when $\beta_{1}<0$ and $\beta_{2}>0$, it indicates a U-shaped relationship between *ER* and *GTFP*; when $\beta_{1}>0$ and $\beta_{2}<0$, it implies an inverted U-shaped relationship. When $\beta_{1}$ and $\beta_{2}$ are both significant, the turning point of the U-shaped or inverted U-shaped curve can be obtained from Equation (4).

(4) $$ER_{tp}=-\frac{\beta_{1}\left(\mu_{i},\;\nu_{i},\;t_{i}\right)}{2\times \beta_{2}\left(\mu_{i},\;\nu_{i},\;t_{i}\right)}$$

### 3.2. GTFP Evaluation Approach

To obtain the GTFP scores of each Chinese city, in this study we adopt the Global Malmquist Luenberger (GML) index [41,42], introduced as:

(5) $$\begin{array}{l} GML_{g}\left(x^{t+1},y^{t+1},b^{t+1},x^{t},\;y^{t},b^{t}\right)=\frac{E^{g}\left(x^{t+1},y^{t+1},b^{t+1}\right)}{E^{g}\left(x^{t},y^{t},b^{t}\right)}=\frac{E^{t+1}\left(x^{t+1},y^{t+1},b^{t+1}\right)}{E^{t}\left(x^{t},y^{t},b^{t}\right)}\left(\frac{E^{g}\left(x^{t+1},y^{t+1},b^{t+1}\right)}{E^{t+1}\left(x^{t+1},y^{t+1},b^{t+1}\right)}\times \frac{E^{t}\left(x^{t},y^{t},b^{t}\right)}{E^{g}\left(x^{t},y^{t},b^{t}\right)}\right)=\\ \;\;\;\;\;\;\;\;\;\;\;\;\;EC\times \left\{\frac{BPG^{g,t+1}\left(x^{t+1},y^{t+1}\right)}{BPG^{g,t}\left(x^{t},y^{t}\right)}\right\}=EC\times BPC \end{array}$$

where *GML* is used to represent the GTFP score in the present study $;\;E^{t}\left(x^{t},\;Y^{t}\right)$ denotes the efficiency change index in year *t*; $E^{g}\left(x^{t},\;Y^{t}\right)$ is the output distance function; *BPG* is a Best Practice Gap between global benchmark technology; $T^{g}$ and $T^{s}$ measures along rays (*x*^s^, *y^s^*), *s* = *t*, *t +* 1; $x^{t},\;y^{t},\;b^{t}$ denotes inputs, desirable outputs, and undesirable outputs of year *t*, which are summarized in Table 1. In addition, if *GML* > 1, it means GTFP growth, whereas if *GML* < 1, GTFP decline is indicated.

### 3.3. Environmental Regulation and Control Variables

#### 3.3.1. Environmental Regulation

The environmental regulation variable has been discussed extensively in empirical studies. In this research, we followed Shen et al. [43] by applying a linear weighted sum method to build a comprehensive index of environmental regulation. The index is based on four indicators, specifically, two pollutants, sulfur dioxide emissions and industrial smoke (dust), and removal rates of two pollutants. The calculation of the environmental regulation variable is a three-step process.

The first step is to standardize the two indicators of the removal rates of sulfur dioxide and smoke (dust). The standardization equation is expressed as:

(6) $$PT_{ij}^{s}=\frac{P_{ij}-\mathrm{Min}\left(P_{j}\right)}{\max\left(P_{j}\right)-\mathrm{Min}\left(P_{j}\right)}$$

where $P_{ij}$ denotes the original removal rate of pollutant *j* in city *I*, while $P_{ij}^{s}$ represents the standardized values; min and max denote minimization and maximization, respectively.

The second step is to set weights for the two pollutants through an adjustment coefficient, $A_{ij}$. The calculation method of $A_{ij}$ is introduced as:

(7) $$A_{ij}=\frac{P_{ij}}{\Sigma_{i}p_{ij}}/\frac{GUP_{i}}{\Sigma_{i}GUP_{i}}$$

where the numerator and denominator denote the ratio of pollutant *j* in city *I* to the total of all cities and the ratio of Gross Urban Product (GUP) in city *I* to the total of all cities. The underlying assumption is that, for two cities with the same pollutant removal rate, the one with higher emissions needs stricter environmental regulation and has a higher weight.

The third step requires us to obtain the stringency of environmental regulation of each city based on the normalized values and the adjustment coefficients obtained from Equations (6) and (7). It can be written as:

(8) $$ER_{i}=\Sigma_{j=1}^{2}A_{ij}PT_{ij}^{s}/2$$

#### 3.3.2. Control Variables

Control variables are added to avoid the problem of omitted-variables bias; they include innovation level (*Innov*), industrial structure (*Second*), urbanization level (*Urban*), economic level (*Econ*), and education level (*Edu*).

Innovation level (*Innov*): refers to how advanced science and technology can help increase energy use efficiency and reduce the intensity of pollutant emissions. Innovation can also promote the upgrading of industrial structures and improve the resource allocation efficiency of enterprises. Therefore, the improvement of the innovation level is conducive to the improvement of GTFP [22]. In our study, the innovation level of a city is measured by the proportion of expenditure on science and technology to GUP.

Industrial structure (*Second*): GTFP growth implies that cities strive to increase output while at the same time minimizing pollution emissions. For cities with low industrial levels, the increase in the proportion of the secondary industry is conducive to the optimization of the industrial structure and productivity gains [36]. However, increases in industrial pollutant emissions caused by rapid industrialization will inhibit GTFP improvements. For GTFP improvements to be realized, industrial structure upgrade and optimization should be urgently advocated. Since the industrial structure of different cities varies significantly from one city to another, heterogeneous impacts on GTFP may exist. In this study, the industrial structure is expressed by the proportion of the secondary industry.

Urbanization level (*Urban*): At the initial stage of urbanization, energy demand expands rapidly due to population aggregation and urban construction leading to rapid increases in various pollutant emissions. As the urbanization rate increases, industrial structure optimization, the agglomeration effect, and the scale effect will improve the quality of economic development [20]. Hence, in the later development of urbanization, more attention will be paid to high-quality development. Therefore, for different cities in different periods, spatiotemporal heterogeneity should exist in the impact of urbanization level on GTFP. The urbanization level was measured in this study by the proportion of land for construction to the total area.

Economic level (*Econ*): At the early stage of economic development, due to the limitations of human capital, technological level, and other factors, pollutant emissions caused by industrialization and urbanization worsen the environment. However, when the economic level develops to a certain stage, local governments and enterprises are inclined to promote technological innovation so that GTFP can be improved. Whereas the Environmental Kuznets curve holds that the relationship between per capita income and environmental pollution shows an inverted U-shaped curve. When per capita income exceeds a certain turning point, the environmental quality can be improved as income continues to increase. Gross urban product per capita (GUP) was used in this study to measure the economic development level of the city.

Education level (*Edu)*: The purpose of education investment is to accumulate human capital from the long-run perspective. On the one hand, GTFP growth may be impaired in the short run because human capital cannot be accumulated overnight and education investment cannot receive returns immediately. On the other hand, investment in education could be used at the outset to introduce advanced technologies, stimulate industrial upgrading, and promote technological progress. However, in the long run, education input plays an important role in improving the ability of the labor force to master new technologies and cultivate talent able to develop advanced technologies, thus promoting the growth of GTFP [34]. In this study, the education level of a city is measured by education expenditure.

### 3.4. Data Sources

When assessing the GTFP scores of Chinese cities, the data for capital stock were calculated using the perpetual inventory method. The data for land for construction and arable land were obtained from Landsat TM/ETM remote sensing images and classified as: land for construction, arable land, forest land, grassland, water area, and unused land. The energy consumption data were computed by processing global nighttime light data. The average annual PM_2.5_ concentrations were obtained from the Atmospheric Composition Analysis Group (https://sites.wustl.edu/acag/datasets/, accessed on 20 December 2020). In addition, the data used to calculate the comprehensive index of environmental regulation and control variables came from the China City Statistical Yearbook (2005–2017) and China Statistical Yearbook for Regional Economy (2005–2017). Any missing data were interpolated. The descriptive statistics of the variables involved in this study, including mean (Mean), standard deviation (S.D.), minimum (Min), and maximum (Max) are given in Table 2.

## 4. Empirical Results

### 4.1. Spatial Distribution of GTFP Scores

**Figure 1:**
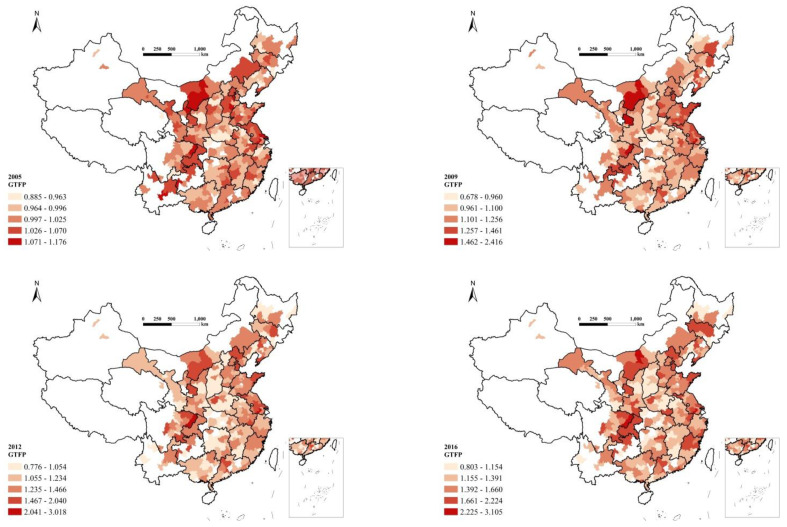
Spatial distribution of GTFP scores of 259 cities in 2005, 2009, 2012, and 2016.

The GTFP scores of 259 cities from 2005 to 2016 were evaluated based on Equation (5) and then mapped with ArcGIS software, as shown in Figure 1.

In general, the spatial distribution of GTFP scores of Chinese cities changed over time. In 2005, the cities with higher GTFP scores were found mainly in the Hohhot-Baotou-Ordos urban agglomeration in Inner Mongolia, the Yangtze River Delta urban agglomeration, and the Sichuan-Chongqing urban agglomeration. In contrast, the cities in the southwestern regions have the lowest scores. Cities in the northeastern and central regions have medium scores. Compared with 2005, the spatiotemporal variations of GTFPs in 2009 changed slightly. However, we noticed GTFP improvements in the Shandong Peninsula and decreases in the Sichuan-Chongqing urban agglomeration. Most of the cities had low GTFP scores in 2012, especially in the central regions. In 2016, the GTFP scores of most Chinese cities improved significantly, especially in the Sichuan-Chongqing urban agglomeration; the implication here is that these cities maintained high-quality development. The GTFP scores of the cities in the southeast also increased greatly. It is noteworthy that the cities in the central region improved, and that the GTFP of most cities has substantially improved in recent years.

### 4.2. Results of the OLS Models

First, we estimated the two-way fixed effects and random effects models. The estimation results of the two models are summarized in Table 3.

We can observe from Table 3 that neither of the coefficients of the variables of *ER* and *ER2* was statistically significant in the random effects model (Model 2). However, in the two-way fixed effects model, namely, Model 1, the coefficients of *ER* and *ER2* were significantly negative and positive, respectively. Subsequently, a Hausman test was used to examine whether the random effects model or fixed effects model would be a better-fitted model; the results support the two-way fixed effects model, indicating that Model 1 was better fitted. From the estimated coefficients of *ER* and *ER2*, we found a U-shaped relationship between environmental regulation and GTFP. In other words, environmental regulation first lowers GTFP and then promotes GTFP improvements. Specifically, at an early stage, the implementation of strict environmental regulation not only increased the environmental protection costs of regulated enterprises, but also decreased GTFP. This is because regulated enterprises have to purchase equipment or adopt clean technology to mitigate pollution emissions, which may increase production costs and impair productivity. On the other hand, the increased environmental investment caused by the requirements of environmental regulation crowded out the productive and profit-making investments of regulated enterprises, which is referred to as a “crowding out effect” [34]. At a later stage, when well-crafted environmental regulations and policies were adopted, regulated enterprises increased environmental investment to utilize green technology, which could partly or fully compensate for the compliance costs. Hence, it allowed regulated enterprises to gain competitive advantages and promote productivity [12]. In addition, to obtain long-term profits, regulated enterprises were also motivated to increase R&D investment to renew production technologies and increase pollution reduction capacities to eliminate the negative externalities of pollution emissions, which are beneficial to GTFP improvements.

As discussed earlier, due to considerable differences in economic level, industrial structure, technological level, etc., among cities, the assumption that the impact of environmental regulation on GTFP in each is fixed may violate spatial heterogeneity. Hence, the GTWR model could be a better choice than the two-way fixed effects model. In addition, we found that the coefficient of determination (R^2^ statistic) of the GTWR model was much higher than that of Model 1. Besides, the Akaike Information Criterion statistic (AIC) of the GTWR model (−0.984) was lower than that (−0.977) of Model 1, indicating that the GTWR model was not only better fitted, but also captured the spatiotemporal heterogeneity of the impact of environmental regulation on GTFP of each city every year.

### 4.3. Impact of Environmental Regulation (ER) on GTFP

**Figure 2:**
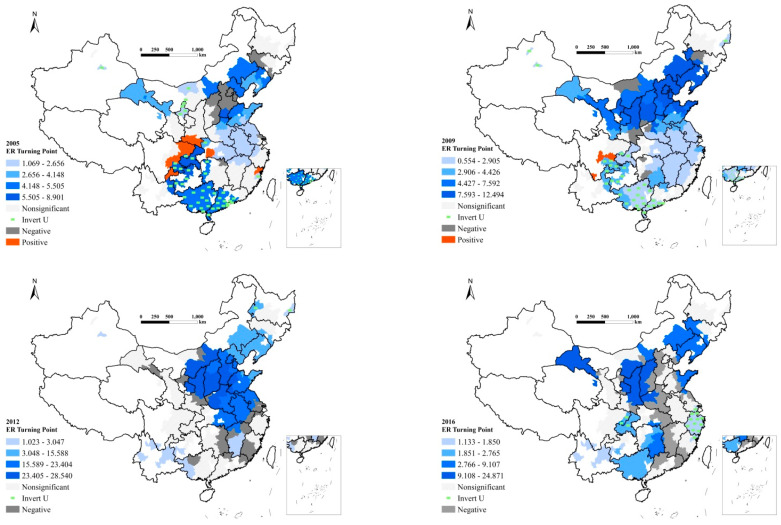
Spatiotemporal distribution of coefficients of environmental regulation in 259 cities.

To better exhibit the spatial heterogeneity of the impact of environmental regulation (ER) on GTFP from 2005 to 2016, we applied the visualization approach to map the estimated coefficients of ER in 2005, 2009, 2012, and 2016, as shown in Figure 2. The maps also provide information on which cities have the turning points of the U-shaped and inverted U-shaped curves between ER and GTFP. Specifically, the blue colors indicate the turning points of the U-shaped curves; importantly, the green areas indicate cities that have an inverted (negative) U-shaped curve between ER and GTFP. The grey (negative) and red areas (positive) indicate that the relationships between ER and GTFP are monotonically increasing and decreasing, respectively. The area in white implies no significant relationship between them in these cities.

By focusing on the cities where the impact of environmental regulation on GTFP shows a linear relationship, we can observe that in 2005, the relationship between environmental regulation and GTFP in several cities such as Guangyuan, Hanzhong, and Ankang decreased monotonically. There were also cities where the relationship between environmental regulation and GTFP was monotonically increasing, e.g., Beijing and its surrounding cities. From 2005 to 2016, fewer cities had positive relationships and more cities had negative relationships. When examining Figure 2, one can observe that in 2016 there were no cities where the relationship was monotonically increasing; the implication here is that, in recent years, environmental regulation has been continuously strengthened in China. As a result, the number of cities where environmental regulation inhibits GTFP has been increasing, indicating that environmental regulation exhibits a negative impact on GTFP improvements.

We also notice a few cities, specifically Beijing, Tianjin, and their surrounding cities in the Hebei province, have greatly changed in the relationship between environmental regulation and GTFP. In these cities, the relationship was at first negative but then turned U-shaped, and finally became negative or nonsignificant once again. Moreover, during the study period, the intensity of environmental regulation in a few cities decreased. For example, the environmental regulation of Baicheng of Jilin province fluctuated only slightly from 2005 to 2009; meanwhile, the GTFP score did not grow significantly either. When environmental regulation intensity was lowered from 2010 to 2016, GTFP had a significant growth trend, implying that strict and inflexible environmental regulation could produce negative effects on GTFP and impair high-quality development. Notably for economically underdeveloped regions, the first priority is to develop the local economy and increase income levels. Hence, relatively lax environmental regulation is conducive to local economic development.

Next, we attend to the cities where the impact of environmental regulation on GTFP shows a nonlinear relationship. In 2005, most cities with an inverted U-shaped relationship between environmental regulation and GTFP were in southern and southwestern China. Specifically, 145 of the total 259 cities had both significant *ER* and *ER2* variables, indicating U-shaped or inverted U-shaped curves. There were 45 cities with an inverted U-shaped curve between environmental regulation and GTFP, and only three cities passed the turning point of the inverted U-shaped curve. In other words, for those three cities, if the government continues to strengthen environmental regulation, GTFP will be inhibited because regulation plays a negative role in GTFP growth when passing the turning point. Cities with a U-shaped relationship between environmental regulation and GTFP were found primarily in the North China Plain. However, most had not passed “Porter’s turning point”. In other words, the negative impact of environmental regulation on GTFP can be explained by “compliance cost”. Specifically, only three out of 100 cities with an inverted U-shaped relationship exceeded the turning point and entered the positive effect stage of “compliance costs”.

For 2009 (Figure 2), the spatial distribution was similar to 2005. Cities with an inverted U-shaped relationship between environmental regulation and GTFP were still mainly found in southern and southwestern China. In addition, among the cities with a U-shaped relationship, the turning points of the cities in North China Plain were generally higher than that of the cities in the Yangtze River Delta urban agglomeration. One likely explanation is that most of the heavy industrial bases were situated in the North China Plain and northeastern China. This finding also implies there are huge differences in industrial structure among Chinese cities. Hence, environmental regulation needs to be reasonably designed and implemented in accordance with the specific conditions of different cities.

In 2012, cities with an inverted U-shaped curve were hardly found. Most cities had U-shaped curves and were mainly concentrated in Liaoning, Beijing, Tianjin, Hebei, Shandong, Shanxi, Henan, Anhui, and Jiangsu provinces. In 2016, the number of cities with a U-shaped curve decreased while the number of cities with a monotonically decreasing relationship was increasing, indicating that environmental regulation in these cities harmed GTFP. Furthermore, in 2016, the cities of the Yangtze River Delta had an inverted U-shaped relationship between environmental regulation and GTFP, indicating that, for these cities, the stricter the environmental regulation, the more likely that high-quality development is undermined.

Since the implementation of the 9th Five-Year Plan (1996–2000), the Chinese government has paid increased attention to environmental protection and taken action for energy conservation and emissions reduction. Most importantly, different environmental regulation targets have been set to abide by specific conditions of different regions. For example, the Yangtze River Delta has the strongest environmental regulation due to its highly developed technology and optimal industrial structure. Correspondingly, the environmental quality in this region has improved markedly. However, if local governments continue to impose stronger regulation, it may begin to negatively affect GTFP; that is to say, hinder high-quality development. Therefore, the level of environmental regulation should not only adapt to local conditions but should also be adjusted in line with specific regional development levels to balance the relationship between economic growth and sustainable development.

### 4.4. Impacts of Control Variables on GTFP

**Figure 3:**
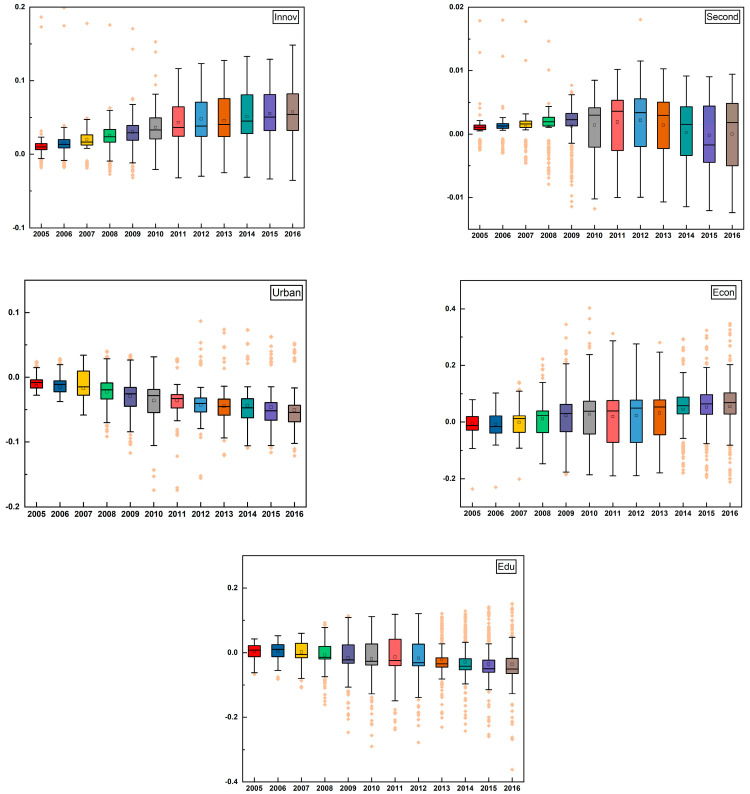
Box plots of the estimated coefficients of control variables from 2005 to 2016.

In addition to the environmental regulation variable, we can also report the estimated coefficients of the control variables. After deleting the insignificant coefficients, the data were plotted in Figure 3 to display the trend of the estimated coefficient of each control variable over time.

Innovation (*Inno*) is regarded as an important means of improving GTFP. In the present study, the impact of innovation on GTFP was almost positive during the sample period. In other words, in general, innovation was conducive to the improvement of GTFP. However, the coefficients of some cities with higher expenditures in science and technology, such as Beijing, and Shenzhen, were lower than that of Urumqi and Baoshan. A credible interpretation is that the marginal elasticity of expenditure on science and technology decreased, given that these developed cities had the highest number of technologies. In other words, expenditure could have been used for other factors enhancing GTFP. Conversely, underdeveloped cities demand greater expenditures in order to increase their technological levels in a bid to improve GTFP.

The impact of industrial structure (*Second*) on GTFP changed strikingly over time. We noticed that the average of the coefficient first increased, then decreased during the sample period. After 2010, the impact of industrial structure on GTFP in most cities was found to be negative, indicating that the high ratio of the secondary industry remained the key factor hindering GTFP growth. In contrast, we observed that the coefficient in several cities changed substantially, e.g., Daqing. At the early stage of the sample period, the ratio of the secondary industry accounted for more than 80%. Thus, it had a negative impact on GTFP. As the ratio declined, the negative impact became insignificant. To conclude, an upgraded and optimized industrial structure is of great significance to high-quality economic development.

The impact of urbanization (*Urban*) on GTFP was basically negative throughout the study period. Moreover, the negative coefficients presented a decreasing trend, suggesting that rapid urbanization may impair GTFP improvement. A reasonable explanation for this is that large amounts of energy-consuming products such as steel and cement have been consumed during urbanization; these processes produce environmental pollution and thus reduce GTFP gains. Whereas we observe that the dispersion of the estimated coefficients changed slightly; this indicated that the impacts of urbanization on GTFP for most cities were similar. However, the downward trend reversed after 2014. One possible reason for this is that, since the National New Urbanization Planning was put forward, China has paid more attention to energy efficiency improvement and pollution emissions reduction in the process of urbanization.

From 2005 to 2006 the impact of the economic level (*Econ*) in most cities on GTFP was negative because the raw resource-dependent economic growth mode at the early stage consumed large quantities of natural resources, including fossil energy, causing serious environmental pollution and impaired GTFP. From 2007 to 2013 the average of the coefficients showed an upward trend, indicating a gradual strengthening of environmental protection and gradual disposal of pollution emissions. From 2014 to 2016, the impact in many cities became positive; nevertheless, China being an enormous country, meant that major spatial differences have remained.

At the early stage of the sample period, one-half of the sample cities had a positive impact of education level (*Edu*) on GTFP, while the others were negative. However, after 2013, the impact of education level on GTFP became negative in most cities. We found that cities with negative coefficients were primarily distributed in Inner Mongolia, Heilongjiang, and Jilin provinces. The underlying reason could be a so-called “brain drain” in recent years, whereby much of the labor force has migrated to developed regions. In contrast, cities with positive impacts were mainly located in Sichuan and Guangdong provinces, indicating that the education level of these regions was conducive to local GTFP growth.

## 5. Conclusions and Policy Recommendations

In this study, we first constructed a comprehensive index of environmental regulation based on the linear weighted method, then adopted the GML index to assess the GTFP scores of 259 Chinese cities, and lastly employed the GTWR model to quantify the impact of environmental regulation on GTFP. The main conclusions and policy recommendations are as follows.

At the early stage of the sample period, in 2005, there were only a small number of cities where the impact of environmental regulation on GTFP showed a linear relationship. In 2016, we noticed that the number of cities was on the rise, showing a monotonically decreasing relationship, indicating that environmental regulation had a positive impact on GTFP. It is noteworthy that most cities were found to have a nonlinear relationship. In 2005, more cities showed a U-shaped relationship than did not. Furthermore, the majority were plotted on the left of the turning point, indicating a negative effect resulting from environmental regulation, that is to say, compliance cost. We found that most cities were in the North China Plain. In addition, among the cities with a U-shaped relationship, there was a significant difference in the turning points of environmental regulation. Conversely, there were fewer cities with an inverted U-shaped relationship as most were on the left of the turning point, thus supporting the Porter hypothesis; these cities were mainly concentrated in southern and southwestern China. In the period between 2005 to 2016, the number of cities with a U-shaped relationship or an inverted U-shaped relationship was decreasing. Furthermore, the relationship in these cities became monotonically decreasing or statistically insignificant.

With profound regional differences among cities, it is necessary to consider local conditions when formulating and implementing policies and measures for the realization of high-quality development targets. Industrial structure plays a core role in promoting economic growth and affecting GTFP. Heavy industry has dominated the North China Plain region for many decades, with large amounts of fossil energy being consumed, culminating in serious environmental pollution and GTFP losses. When stricter environmental regulation is implemented, GTFP will rapidly decline because industrial output values will greatly decrease, despite that industrial pollutant emissions are reduced. Therefore, an effective and efficient way to reduce environmental pollution emissions and improve GTFP in this region is to engage in technological progress and industrial technology upgrades.

We observed that the GTFP scores of most cities in southwestern China were relatively high, which was closely linked to improved environmental quality. However, high scores also indicate underdeveloped economic levels due to lower industrialization. Hence, environmental regulation has no negative impact on local high-quality development. Different from the developed eastern coastal region, southwestern cities may have to rely heavily on industrialization to increase their local economic levels, but not necessarily at the expense of the local environment. Therefore, it is urgent to attract hi-tech and advanced industries to the southwest through preferential industrial policies. Importantly, however, environmental regulation measures are still necessary because environmental quality is one of the essences of high-quality economic development; having a higher economic level and a cleaner living environment are principal values to local people. As it is the role of central and local governments across China to combat the negative externalities of environmental pollution and coordinate the interests of all parties in the process of the economic transition, various levels of local governments have gradually adopted environmental regulation.

Our findings for 2016 showed an inverted U-shaped curve in the cities of the Yangtze River Delta, indicating that stricter environmental regulation may inhibit high-quality development. The Yangtze River Delta has long been the leading economy among Chinese cities, its hallmarks being a high economic level, faster technological progress, and optimal industrial structures. However, if and when stricter environmental regulation is imposed, it will impair GTFP in the short run. A better way to solve the problem would be to transfer energy-intensive and highly polluting industrial sectors, along with fairly high-level technologies and advanced equipment from the Yangtze River Delta region, to the energy-abundant west, which is likely not only to reduce environmental pollution within the region, but also promote economic growth in the west.

Compared with other studies in the existing literature, the merits of our research are twofold. One is that we considered a comprehensive index of environmental regulation, while other studies have usually adopted the removal rate of a single pollutant. In this sense, the index of environmental regulation in our study can fully capture the measure of stringency of environmental regulation. The other is that we adopted the GTWR model to quantify the spatiotemporal heterogeneity of the impact of environmental regulation on GTFP, while other studies have considered the fixed relationship between the two variables in a specific period. Indeed, linear or nonlinear relationships could also vary with time. In this sense, our findings may provide a novel insight into the investigation of the time-varying relationship between environmental regulation and GTFP. Nevertheless, our study may have two shortcomings. One is that we fail to extend our data to the latest year due to the data unavailability of some key variables. Hence, future research could update these data or replace them with other data. The second shortcoming is that we did not include more explanatory variables due to data unavailability at the prefecture level. Hence, future work should incorporate other important factors and have an in-depth discussion about the spatiotemporal heterogeneity impacts on GTFP.

## Figures and Tables

**Table 1:** Indicators for GTFP evaluation.

Factors		Indicators	Unit
Inputs	Land	Land for construction	square kilometer
		Arable land	square kilometer
	Resources	Energy consumption	Ton
		Total urban water supply	ten thousand tons
	Capital	Total investment in fixed assets	ten thousand yuan
	Labor	Number of employees	ten thousand employees
Outputs	Desirable output	Gross urban product	ten thousand yuan
	undesirable outputs	Industrial wastewater discharge	ten thousand tons
		Annual average PM_2.5_ concentrations	μg/m^3^

**Table 2:** Definitions and descriptive statistics of variables involved in this study.

Variable	Definition	Unit	Mean	S.D.	Min	Max
GTFP	GML index	Na.	1.173	0.245	0.678	3.105
*ER*	Environmental regulation by comprehensive evaluation method	Na.	1.179	1.590	0.001	39.782
*Innov*	Ratio of expenditure on science and technology to GUP	%	7.671	40.398	0.001	1061.374
*Second*	Ratio of the secondary industry to GUP	%	49.800	10.370	9.000	90.970
*Urban*	Ratio of land for construction to total area	%	0.072	0.071	0.002	0.483
*Econ*	GUP per capita	Yuan	35,586.9	27,637.7	2396.1	256,889.2
*Edu*	Education expenditure	million Yuan	41.124	55.065	0.099	887.368

**Table 3:** Results of fixed effects and random effects models.

Variables	Model 1 Two-Way Fixed Effects	Model 2 Random Effects
*ER*	−0.0118 ***	−0.00341
	(0.00268)	(0.00258)
*ER2*	0.000231 ***	1.89 × 10^−5^
	(8.91 × 10^−5^)	(8.87 × 10^−5^)
*Lninnov*	0.0160 ***	0.0312 ***
	(0.00443)	(0.00356)
*Second*	−0.000115	−0.00279 ***
	(0.000448)	(0.000370)
*LnUrban*	0.0905 ***	−0.0239 ***
	(0.0170)	(0.00688)
*LnEcon*	−0.0122	0.0989 ***
	(0.0154)	(0.00987)
*LnEdu*	0.0124	0.0279 ***
	(0.0119)	(0.00666)
*Constant*	0.316	−1.151 ***
	(0.192)	(0.0736)
*Obs.*	3108	3108
*R* ^2^	0.613	-

Note: Standard errors in parentheses; ***: *p* < 0.01.

## Data Availability

The data presented in this research are available on request from the corresponding author.

## Statement

This article is in memory of the second author, Chen Yuan, who passed away on the 30 December 2022 at the age of 25. He is a very good person, friend and student of ours.
